# Unique clinical, morphological, and molecular characteristics of tumors associated with PSC-IBD

**DOI:** 10.1007/s00428-025-04072-y

**Published:** 2025-03-19

**Authors:** Andrea Vajsova, Monika Cahova, Lukas Bajer, Eva Sticova, Ivana Juskova, Mojmir Hlavaty, Ondrej Fabian

**Affiliations:** 1 Clinical and Transplant Pathology Centre, Institute for Clinical and Experimental Medicine, Prague, 14021 Czech Republic; 2 Institute of Pathology of the First Faculty of Medicine and General Teaching Hospital, Prague, 12800 Czech Republic; 3 Experimental Medicine Centre, Institute for Clinical and Experimental Medicine, Prague, 14021 Czech Republic; 4 Department of Gastroenterology and Hepatology, Institute for Clinical and Experimental Medicine, Prague, 14021 Czech Republic; 5 Department of Internal Medicine, Second Faculty of Medicine, Charles University, Prague, 15000 Czech Republic; 6 Department of Pathology, Royal Vinohrady Teaching Hospital, Srobarova 1150/50, Prague, 10000 Czech Republic; 7 Department of Pathology and Molecular Medicine, 3rd Faculty of Medicine, Charles University and Thomayer Hospital, Prague, 14059 Czech Republic

**Keywords:** Primary sclerosing cholangitis, Inflammatory bowel disease, Ulcerative colitis, Crohn’s disease, Colorectal carcinoma, Cholangiocarcinoma

## Abstract

Primary sclerosing cholangitis (PSC) is a rare cholestatic liver disease characterized by chronic inflammation and progressive fibrosis of the biliary tree, leading to significant liver function impairment over time. There is a strong association with inflammatory bowel diseases (IBD), together representing a distinct and complex medical condition. Patients with PSC-IBD face a heightened risk of various cancers, particularly colorectal carcinoma (CRC) and cholangiocarcinoma (CCA) as the most common types. In this review, we aim to characterize the distinctive features of PSC-IBD-associated carcinomas. Cancer pathogenesis in PSC-IBD is shaped by various factors including dysregulated bile acid metabolism, gut dysbiosis, and unique immune responses. PSC-IBD-associated CRC is often right-sided and warrants vigilant monitoring due to its higher incidence and unique morphological features compared to CRC arising in the terrain of IBD alone. CCA shares substantial genetic similarities with extrahepatic CCA and poses diagnostic challenges since it is frequently detected at advanced stages due to symptom overlap with PSC. Besides, reliable predictive biomarkers for targeted therapy remain largely unexplored. The distinct molecular, genetic, and histopathological profiles of CRC and CCA in PSC-IBD underscore the complexity of these malignancies and highlight the need for continued research to develop precise therapeutic strategies.

## Introduction

Primary sclerosing cholangitis (PSC) is a rare cholestatic liver disease of unknown etiology, characterized by chronic inflammation of the extrahepatic and large intrahepatic bile ducts, leading to progressive fibrosis of the biliary tree. This fibrotic process results in ductal narrowing and obstruction, causing chronic cholestasis and subsequent hepatic injury. PSC is more prevalent and often more severe in males (male-to-female ratio of about 2: 1), with an average diagnosis age of 30–40 years [1]. Approximately 60–80% of patients with PSC have concomitant IBD [2]. PSC associated with IBD (PSC-IBD) represents a unique and complex medical condition that poses significant challenges for patients. Patients with the PSC-IBD phenotype exhibit demographic characteristics similar to those seen in PSC cases; however, the diagnosis of PSC typically occurs at a younger age in these individuals compared to those with PSC alone [3]. Although PSC-IBD is now recognized as a distinct entity and often reported as IBD without any further subclassification, the intestinal disease can in fact resemble either ulcerative colitis (UC), which is the more common form, or present with features closer to Crohn’s disease (CD). In the studies discussed below, we have maintained the distinction between UC and CD to reflect the terminology used in the original study texts. In the minority of PSC cases, only the small intrahepatic bile ducts are affected, termed small duct PSC, which usually shows a milder course and better prognosis, but may progress to large duct disease [4]. PSC-IBD tends to cause more intense inflammation in the right colon [3, 5], unlike the typical pattern of UC, which worsens towards the distal end. Some studies found higher occurrences of rectal sparing, backwash ileitis, right-sided activity predilection, and pancolitis in PSC-IBD patients compared to matched UC controls [6, 7].

The PSC patients in general face significantly higher risk of developing cancer including hepatobiliary, colorectal, pancreatic, gastric, and small bowel carcinomas or various lymphomas [8]. Among these, the most common cancers developing in this context of PSC-IBD are colorectal carcinoma (CRC) and cholangiocarcinoma (CCA). CRC is a major complication in PSC-IBD patients and has been shown to occur at a higher rate and exhibit distinct characteristics compared to individuals with IBD alone. Colitis-associated CRC constitutes roughly 1% of all CRC cases [9]. Studies have highlighted the challenges in detecting colitis-associated neoplasia, often presenting as flat and difficult-to-visualize lesions during endoscopy [10]. The combination of chronic inflammation, oxidative stress, microbial dysbiosis, and immune responses contributes to the complex pathogenesis of CRC in PSC-IBD patients. Biliary tract carcinoma (BTC) is a heterogeneous group of tumors that poses significant challenges in identifying a precise mechanism of cancerogenesis. Numerous hypotheses have been proposed to explain the onset and progression, but no single theory can account for all cases, with disrupted bile acid homeostasis, inflammatory processes, and bile duct damage probably playing an important role. In the context of PSC-IBD, the complexity is further amplified. Incidence of CCA among patients with PSC is variable, ranging from 4 to 36%, and that the risk is particularly elevated shortly after PSC diagnosis, with 30–50% of CCA cases identified within the first year [11]. CCA represents the most prevalent biliary tract tumor and the second primary hepatic malignancy, encompassing intrahepatic (iCCA) and extrahepatic (eCCA), the latter including perihilar (pCCA), and distal (dCCA) subtypes.

The purpose of this review is to comprehensively characterize the distinctive clinical, morphological, and molecular features of large bowel and biliary tract carcinomas arising in the context of PSC-IBD and to highlight their differences from sporadic cases.

## Colorectal carcinoma

### Pathogenesis

Colorectal carcinoma (CRC) is a major complication of patients with PSC-IBD, and it has been reported in many cohort studies that PSC-IBD patients harbor an increased risk of CRC, with PSC being a strong independent risk factor [12, 13]. The presence of chronic inflammation, its severity, and duration seem to be a major known contributing factor in cancerogenesis of colitis-associated CRC generally. Analysis of Rutter et al. [14] performed on 68 patients with UC showed a significant correlation between the colonoscopic and histological inflammation scores and the risk of CRC. Inflamed colonic mucosa shows deviations in major carcinogenic pathways that lead to CRC, even prior to any histological indications of dysplasia or cancer being evident. Oxidative stress induced by prolonged inflammation likely plays a role, with signs of oxidative harm and DNA double-strand breaks escalating progressively from inflammation to dysplasia and ultimately to carcinoma [15, 16]. Role of the important transcription factor NF-kappa B (NF-κB) in the cancerogenesis of IBD-associated CRC has been highlighted in review by Schottelius et al. [17]. NF-κB plays a dual role; it regulates cytokine expression and inflammatory processes in IBD and promotes tumor cell proliferation and survival by controlling antiapoptotic genes. Constitutive NF-kB activation in CRC tissue correlates with tumor progression and may influence both colitis-associated and sporadic CRC (sCRC) through its connections to angiogenesis and chemotherapy resistance. It is important to note that inflammation, considered independently, may manifest differently in PSC-IBD compared to IBD alone or other inflammatory conditions in the colon, necessitating targeted research to understand the differences between these processes. PSC-IBD exhibits unique inflammatory traits that differentiate it from other inflammatory conditions. Study by Shaw et al. [18] identified unique adaptive inflammatory transcriptional signature associated with greater risk and shorter time to dysplasia in patients with PSC-IBD compared to non-PSC-IBD patients. This inflammatory signature features antigen-driven IL-17A + FOXP3 + CD4 T cells with a pathogenic IL-17 profile and an increase in IgG-secreting plasma cells. Wittek et al. [19] found that most PSC patients, even those without clinical signs of IBD, had immune cell infiltration and higher IL17A and IFNG expression in intestinal biopsies. Subclinical inflammation in PSC patients was localized in the distal colon, while PSC-IBD patients had inflammation in either the distal colon, the right colon, or the terminal ileum.

The strong connection between IBD and PSC has highlighted the gut microbiota as a key factor in the development of PSC and contribution to cancer development through interactions with the host’s immune system, production of carcinogenic metabolites, and impact on inflammation. An increasing number of studies have suggested a link between dysbiosis of the gut microbiota and CRC [20, 21]. In the study by Kummen et al. [22] patients with PSC exhibited significantly lower bacterial diversity and distinct microbial composition compared to healthy controls and UC patients, showing 11 reduced genera and an increased Veillonella genus, which is associated with chronic inflammation and fibrosis, regardless of IBD status. Apart from that, mycobiota, particularly specific *Candida* species found in tumor samples across various gastrointestinal sites, are increasingly recognized as a potentially impactful component of the human microbiome in CRC pathogenesis [23].

Gut inflammation can lead to liver inflammation through mechanisms such as increased intestinal permeability (“leaky gut”) and the subsequent migration of bacteria and toxins to the liver and bile ducts potentially contributing to development of cancer. This topic, regarding gut-liver axis, is further discussed in the corresponding section about biliary tract cancer.

Bile acids (BAs), liver-synthesized cholesterol derivatives, are crucial not only for lipid absorption, but also as potent signaling molecules. They have also been identified as potent carcinogens in CRC. BAs contribute to CRC development, damaging colonic epithelial cells and inducing oxidative stress and genomic instability, while promoting progression through mechanisms like apoptosis inhibition and enhanced proliferation, invasion, and angiogenesis. Key signaling pathways, such as those involving the EGFR and associated cascades, are activated by bile acids, affecting various genes involved in cancer growth [24].

### Clinical presentation

Hence, the previously discussed inflammatory pattern, CRC associated with PSC-IBD, is more likely to occur in the proximal colon and is identified at a more advanced stage [25], with poorer survival compared to IBD alone [25]. Patients with PSC-UC exhibit significantly more subclinical endoscopic and histologic activity in the right colon and greater histologic inflammation in the proximal colon compared to non-PSC-IBD patients, even during clinical remission, resulting in unnoticed prolonged inflammation [26].

In contrast to sporadic CRC where precursor lesions are usually polypoid and visible via endoscopy, colitis-associated CRC presents a surveillance challenge. Dysplastic lesions in colitis are often flat and not easily seen during endoscopy, and while dyes have traditionally been used to enhance visualization [10], this practice has largely been replaced in clinical settings by virtual chromoendoscopy. Multifocality is another feature differentiating colitis from the more localized patterns seen in sporadic cases [27]; importantly, in PSC-IBD, the practice of performing random biopsies during colonoscopy should be actively maintained to ensure thorough assessment.

Typical symptoms of right-sided CRC, such as abdominal pain or discomfort in the right quadrant, changes in bowel habits (including diarrhea and constipation), and unexplained weight loss, may contribute to delayed diagnosis due to their nonspecific nature. Patients may also experience fatigue and iron deficiency anemia resulting from chronic bleeding, alongside gastrointestinal disturbances like nausea and vomiting. In advanced cases, symptoms such as a palpable abdominal mass or evidence of bowel obstruction may be observed. The increased prevalence of inflammation in the right colon in PSC-IBD patients highlights the importance of vigilant screening and monitoring for early detection of CRC, specifically, there is a need for annual colonoscopy in those with diagnosed PSC-IBD [28].

Managing therapy in PSC-IBD patients with current CRC involves cautious balancing due to the possible negative impact on IBD flares and survival. A systematic review of 33 studies involving 1298 IBD patients undergoing cancer treatment found that 30% experienced flares, with a significant use of systemic steroids and biologic therapies, and a heightened risk of gastrointestinal toxicity from immune-checkpoint inhibitors treatment compared to non-IBD patients, yet these flares were generally manageable and should not prevent appropriate cancer treatment [29]. Meta-analysis Lu et al. [30] found that patients with IBD-associated CRC have significantly worse overall and cancer-specific survival compared to non-IBD CRC patients. PSC-IBD patients, despite similar CRC-related mortality, had significantly lower 5-year survival rates and more advanced tumors compared to those without PSC [25].

### Histopathology

As discussed in the previous study [13], an analysis of a cohort of PSC-IBD patients, with UC phenotype being the most common, revealed that nearly one-third developed dysplasia over a mean follow-up period of 46 months. This dysplasia often exhibited nonconventional features, being frequently invisible to endoscopic/gross examination, right/proximal-sided, multifocal, and more likely progressed to advanced neoplasia. Crypt cell dysplasia and hypermucinous dysplasia were the most common types. Nonconventional dysplasia was often detected in the same colonic segment as CRC or immediately adjacent, with rates similar to conventional dysplasia and was more commonly associated with subsequent high-grade (poorly differentiated) CRC compared to conventional dysplasia [31]. In the study [32] describing morphology of CRC in IBD patients without concomitant PSC, 11 cases were examined, most of which were classified as grade 2 with tubular and cribriform growth. Notably, two cases were well-differentiated (grade 1), two high-grade (grade 3), with some exhibiting gastric mucin marker expression, mucinous carcinoma components, and one case featuring a poorly cohesive/signet ring cell carcinoma. There is a paucity of studies describing the specific morphology of CRC in IBD patients. Some studies suggest [33] that colitis-associated CRCs are characterized by a lack of tumor necrosis, a Crohn-like reaction, tumor histologic heterogenity, the presence of mucin, and signet ring cell differentiation. However, these findings primarily pertain to patients with IBD without concurrent PSC. Various treatments can significantly affect the morphology of dysplasia and carcinoma development in response to treatment-induced stress and immune activation.

### Genetics

Conventional, sCRC, is classified into nonhypermutated and hypermutated groups. Nonhypermutated cancers are usually microsatellite stable (MSS), characterized by chromosomal instability and common mutations in genes such as APC, TP53, KRAS, and many others [34]. Hypermutated cancers often involve microsatellite instability (MSI) due to defective DNA mismatch repair genes, including sporadic cases with MLH1 promotor hypermethylation and inherited cases such as Lynch syndrome. A subset is ultramutated, typically due to DNA polymerase defects.

**Fig. 1:**
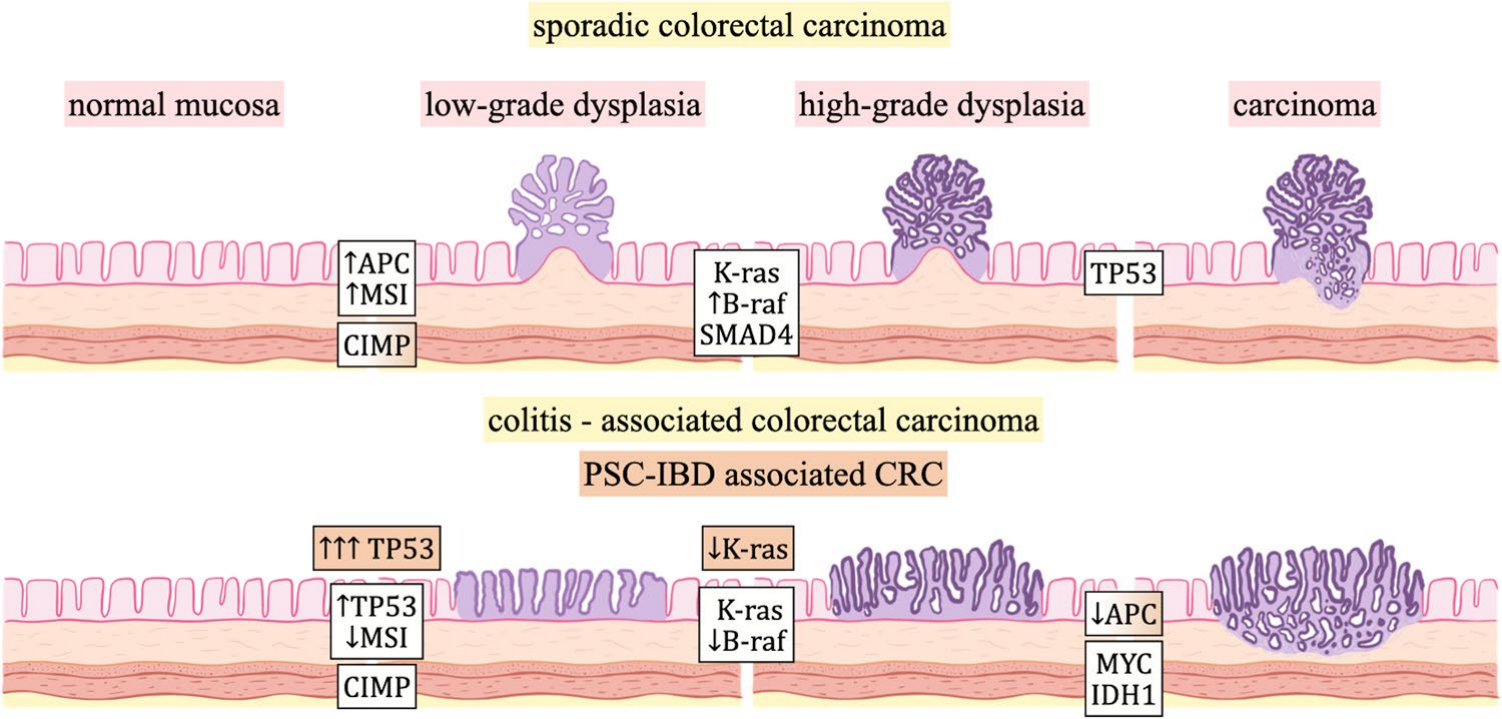
Genetic alterations in sporadic, colitis-associated and PSC-IBD-associated colorectal carcinoma. CRC = colorectal carcinoma, APC = adenomatous polyposis coli, MSI = microsatellite instability, CIMP = CpG island methylator phenotype

Early phases of both sporadic and colitis-associated CRC may feature MSI, CpG island methylation, and microRNA alterations. The most pronounced genes involved are highlighted in Fig. 1. The molecular pathogenesis of colitis-associated CRC, primarily studied in IBD patients without concomitant PSC, differs in the timing and frequency of certain genetic alterations during the dysplasia-carcinoma sequence. Patients with PSC-IBD, as discussed previously, are at higher risk of harboring nonconventional and/or invisible dysplasia, particularly in the right/proximal colon, compared to those with IBD alone. A study Zhang et al. [35] examining DNA content in benign colon biopsies from PSC-IBD patients found a significant presence of DNA abnormalities in the right/proximal colon, even in the absence of dysplasia. This DNA abnormality was notably more common in PSC-IBD patients than in IBD patients without neoplasia. The presence of abnormal DNA content in PSC-IBD patients was frequently followed by the detection of nonconventional and/or invisible dysplasia.

Chronically inflamed colonic mucosa in IBD patients undergoes cancer-associated molecular alterations before dysplasia is histologically evident, indicating a predisposition to multifocal precancerous and cancerous changes [36]. For instance, TP53 mutations occur before histologically apparent dysplasia in colon mucosa, whereas in sCRC they occur in later changes of high-grade dysplasia [37]. In sCRC, mutations in the APC gene are recognized as the initial events in tumorigenesis; however, this does not appear to be the initiating mechanism in colitis-associated CRC [38]. The whole-genome sequencing of colonic crypts from IBD patients revealed that the mutation rate in affected colonic epithelial cells is 2.4 times higher compared to healthy cells from non-IBD controls. Nonsynonymous mutations were discovered in ARID1A, FBXW7, PIGR, and ZC3H12A genes, and in interleukin 17 and Toll-like receptor pathways, indicating distinct selection mechanisms in the colitis-affected colon [39]. In colitis-associated CRC, MYC amplifications and IDH1 mutations are more frequent compared to sCRC. IDH1 mutations were significantly enriched in patients with a history of CD versus UC. Conversely, BRAF V600E mutations are notably less frequent in colitis-associated CRC than in sCRC [40].

Most studies on colitis-associated CRC focus on patients with IBD irrespective of concomitant PSC, with few investigations specifically addressing the genetics and molecular characteristics unique to PSC-IBD patients. In a study by Krijger et al. [41], the chromosomal aberrations in PSC-IBD-associated CRC were similar to those in IBD-CRC and sCRC, with MSI being rare. Mutation frequencies were largely comparable across the groups, except for lower KRAS mutations in PSC-IBD CRC compared to IBD-CRC and sCRC, and lower APC mutations in PSC-IBD and IBD compared to sCRC. PSC-IBD cases frequently showed CpG island methylator phenotype (CIMP) at similar levels to cases of sCRC, but less than IBD CRC. PSC-UC patients show even higher p53 expression in the nondysplastic mucosa as compared to active UC without PSC [42].

### Predictive molecular biomarkers

RAS-encoded proteins, a family of small GTPase-related proteins, play a crucial role in cell signal transduction. KRAS and NRAS are therapeutically significant, and international guidelines [43] suggest analyzing specific codons that correlate with resistance to anti-EGFR therapies. Nearly 50% of sporadic CRCs harbor RAS mutations and are unsuitable for anti-EGFR treatment, yet only 40–60% of RAS-wildtype cases respond, indicating a complex underlying biology affecting treatment efficacy. Therapies targeting EGFR pathways in IBD may help reduce long-term cancer risk by optimizing the response to chronic inflammation [44]. BRAF mutations, particularly p.V600E, are critical prognostic biomarkers in sCRC, they are associated with adverse outcomes and helpful in excluding Lynch syndrome, given their mutual exclusivity with RAS mutations and the lack of benefit from anti-EGFR therapy for patients with BRAF p.V600E mutations [45]. In BRAF-wildtype cases, MSI indicates a favorable prognosis but reduces the effectiveness of fluorouracil-based chemotherapy, regardless of BRAF status. MSS CRC with BRAF mutations generally have a poor prognosis, while recent studies show that MSI is significant for immunotherapy, as microsatellite-unstable cancers respond well to PD-1 inhibitors in patients who have failed conventional therapies [46]. Regarding tumor-infiltrating lymphocytes (TILs), immune cells found within the tumor microenvironment, the results of meta-analysis Idos et al. [47] demonstrate that high levels of generalized TILs as compared to low levels, had an improved overall survival in sCRC.

Predictive biomarkers in colitis-associated CRC exhibit distinct characteristics compared to sCRC. KRAS mutations, which are less common in colitis-associated CRC, can influence the efficacy of anti-EGFR therapies, while BRAF V600E mutations, typically linked to poor prognosis, are uncommon in this context. Colitis-associated CRC is generally MSS, which diminishes the relevance of MSI as a predictive marker and limits the role of TILs, as these tumors do not typically exhibit the immune response found in MSI-H tumors. Additionally, IDH1 mutations may be more prevalent in colitis-associated CRC and could indicate unique metabolic and epigenetic changes. The potential use of immune checkpoint inhibitors targeting PD-L1 requires further investigation. Unlike T cells present in sCRC, TILs do not seem to improve the prognosis of colitis-associated CRC [48]. This might be associated with a reduction in granzyme B expression within these tumors. Additionally, it is important to note that studies typically do not distinguish between colitis-associated CRC without PSC and with PSC, making it challenging to find information specifically on predictive markers in PSC-IBD-associated CRC. This distinction is crucial for fully understanding the predictive characteristics and treatment responses unique to PSC-IBD patients.

## Biliary tract carcinoma

### Pathogenesis

The development of cholangiocarcinoma (CCA) in patients with PSC-IBD might differ from the mechanisms underlying CCA that occurs without PSC. In PSC-IBD, impaired bile acid homeostasis due to obstruction in bile ducts and disrupted enterohepatic circulation may contribute to liver and bile duct damage and retaining a chronic inflammation. This may drive cholangiocytes to proliferation, undergoing various metaplastic changes and subsequent development of dysplasia [49]. When occurring in the context of PSC-IBD, this disruption in bile acid balance may be potentiated by damage to intestinal barrier. CCA originates from transformed cholangiocytes [50], with hepatic progenitor cells potentially being the source. High-grade stricture of the bile duct, defined as a biliary stricture on magnetic resonance imaging / cholangiopancreatography with > 75% reduction of duct diameter in the common bile duct or hepatic ducts [51], may contribute significantly to carcinogenesis in this region, leading to the constant inflammatory damage and repeated episodes of biliary infections. Cyclooxygenase-2 (COX-2) and microsomal prostaglandin E synthase-1 (mPGES-1) are believed to promote carcinogenesis through the production of prostaglandin E2. In a study by Ishii et al. [52] aiming at patients with PSC, COX-2 showed strong expression in both CCA tissues and nonneoplastic bile duct epithelial cells when compared to sporadic CCA. Proinflammatory cytokines, notably IL-6, and their epigenetic regulation of gene expression can promote tumor progression by modifying promoter methylation and affecting the expression of growth-regulatory pathways, including those associated with EGFR. On the other hand, the study by Lieshout et al. [53] that investigated several cytokines related to PSC on organoids including IL-1β, IL-6, IL-17A, interferon gamma, and tumor necrosis factor alpha found that only IL-17A significantly increased cell proliferation and organoid size. IL-17A expression was found to be higher in PSC-associated CCA than in sporadic CCA, correlating with increased tumor proliferation.

T lymphocytes, particularly those marked by gut-homing properties, play a significant role in the pathogenesis of PSC. These cells, including effector T cells that become long-lasting memory cells during gut inflammation, can migrate to the liver via the enterohepatic circulation. They adhere to both the intestinal mucosa and hepatic endothelium through adhesion molecules, with a significant portion expressing the gut-homing integrin alpha4beta7 [54]. Aberrant expression of homing molecules such as MAdCAM-1 in PSC livers enhances the migration of gut-primed T-cells, intensifying inflammation as PSC progresses [55]. MAdCAM-1 and other markers were also elevated in various chronic liver diseases suggesting gut-derived T cell recruitment as a common feature that is not unique to PSC [56].

During gut inflammation, barrier integrity weakens, leading to increased permeability, a condition known as “leaky gut.” This allows microbe-related proinflammatory substances and possibly whole microbes to travel from the gut to the liver through the portal system, triggering hepatic inflammation. In this context, pathogen-associated molecular patterns (PAMPs) may play a crucial role by interacting with Toll-like receptors (TLRs) on liver cells [57]. Ultimately, prolonged IBD duration may be a key factor in elevating CCA risk in PSC-IBD patients, regardless of whether they underwent colectomy [58]. Various studies [22, 59] investigated differences in gut microbiota and analyzed fecal and bile samples, finding differences in microbial composition and biodiversity between PSC, PSC-IBD, and healthy controls. Regarding fungi, some studies revealed a unique fungal microbiota imbalance in IBD and PSC, marked by changes in their diversity and composition [60]. Nevertheless, the causal relationship with PSC-IBD and CCA remains to be proved.

### Clinical presentation

The clinical manifestations of PSC-associated CCA include right upper quadrant abdominal pain, jaundice, cholangitis, fatigue, itching, weight loss, ascites, hepatomegaly, splenomegaly, and abnormal liver function tests [61]. Although these symptoms are common in patients with PSC and are not entirely specific for CCA, sudden worsening of these conditions, particularly changes in liver biochemistry, can suggest malignant transformation [62]. In general, patients with extrahepatic CCA (eCCA) often display symptoms of biliary obstruction, while those with intrahepatic (iCCA) more frequently present with generic cancer-related symptoms.

Diagnosing CCA in the setting of PSC is challenging [1], due to the nonspecific initial symptoms of CCA, overlap in signs and symptoms between PSC and CCA, and the lack of highly sensitive diagnostic methods. Majority of the cases involving CCA associated with PSC manifest with a high-grade stricture, previously referred as dominant stricture (DS) [63]. Imaging techniques such as abdominal ultrasound, computed tomography and/or magnetic resonance imaging with cholangiopancreatography are employed to identify and evaluate DS. Furthermore, endoscopic retrograde cholangiopancreatography (ERCP) is an essential tool in diagnosing CCA by allowing for tissue sampling and even direct visualization when combined with cholangioscopy [64].

In study by Chahal et al. [65] evaluating cancer stages, it was observed that PSC patients, whether with or without IBD, frequently presented at stage 4 with distant metastases. Perihilar tumors were notably prevalent in PSC patients at stage 3. This study highlighted that PSC is predominantly associated with the perihilar (pCCA) subtype. Late-stage diagnosis, attributed to symptom evasion and diagnostic challenges in the presence of PSC, underlines the need for regular surveillance using imaging techniques to enable earlier detection and improved outcomes. Most cases of PSC-associated CCA are diagnosed at an advanced stage making radical surgery and liver transplantation unfeasible with poor prognosis. When tumors are small or discovered incidentally, liver transplantation results in a 35% survival rate over 3 to 5 years [66].

### Histopathology

CCAs can be classified into intrahepatic and extrahepatic types, with iCCA further divided into large duct and small duct subtypes. Large duct iCCA arise in the large intrahepatic bile ducts, often presenting as periductal infiltrating lesions with associated desmoplastic stroma and frequent invasion to portal structures. Histologically, it resembles pCCA and distal (dCCA) [67]. Small duct iCCA, which typically occurs in the hepatic periphery, shows mass-forming growth. Grossly, eCCA may appear as sclerosing, nodular, or papillary lesions, with the sclerosing type being the most prevalent and causing circumferential constrictive thickening of the bile ducts. Microscopically, most sporadic eCCA in general are pancreatobiliary-type adenocarcinomas, characterized by irregular glands, small cell clusters, and a desmoplastic stroma, iCCA show a ductal or tubular pattern with a variable-sized glands.

Study by Carpino et al. [68] comparing samples from normal livers, PSC, and PSC-associated CCA revealed increased inflammation, peribiliary gland activation marked by their increased proliferation, and several other histopathological variables as potential markers in neoplastic ducts. CCA cells displayed epithelial-to-mesenchymal transition traits and lacked primary cilia. The comprehensive analysis by Goeppert et al. [69] of patients with PSC-associated biliary tract cancers (PSC-BTC) revealed a wide range of anatomical locations and histological subtypes. This included iCCA, pCCA, dCCA, gallbladder cancers, and some cases with unknown origins. Over half of the cases displayed uncommon histomorphologies such as papillary, mucinous, solid, diffuse, intestinal, or adenosquamous, rather than the typical ductal or glandular forms. Unique histological patterns, including cholangiolar/small-duct patterns, were identified in some cases. The findings suggested a significant association between PSC-BTC cases and the presence of intraductal or intracystic papillary neoplasms of the bile duct, with the majority linked to invasive CCA. Tumor grading revealed a prevalent occurrence of moderate grades. A key finding of this study is that PSC‐BTC exhibits a phenotype that is characteristic of extrahepatic, large‐duct BTC, independent of the anatomical location of the tumor.

### Genetics

**Fig. 2:**
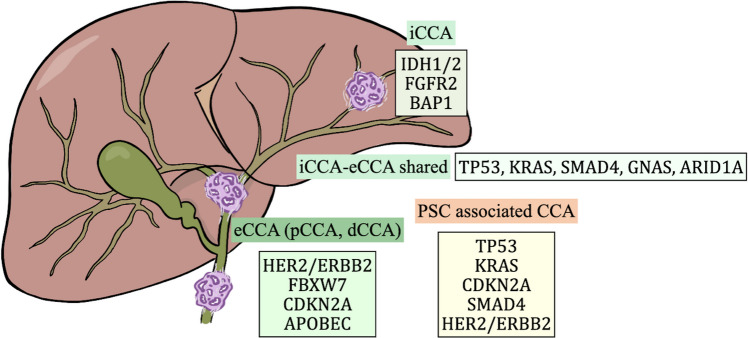
Genetic alterations in iCCA, eCCA and PSC-IBD-associated CCA. eCCA = extrahepatic cholangiocarcinoma; iCCA = intrahepatic cholangiocarcinoma; pCCA = perihilar cholangiocarcinoma; dCCA = distal cholangiocarcinoma; PSC = primary sclerosing cholangiti

In CCA, numerous molecular genetic alterations have been discovered across various tumor suppressor genes and oncogenes. Prior research has demonstrated that these genomic alterations vary according to the anatomical subtype and underlying etiology [70]. Molecular profiling of distinct BTC subtypes reveals specific clusters of genetic changes that group into functional categories, potentially advancing precision oncology approaches (Fig. 2). The molecular and mutational profiles vary across anatomical subtypes of CCA. The most frequent mutation event in CCA in general was observed in the KRAS gene, with other shared mutations in TP53, SMAD4, GNAS, and ARID1A. Sets of genes such as IDH1/2, BAP1 mutation, and FGFR2 fusions were exclusively found in iCCA [70]. Most frequent genetic alterations in eCCA are TP53, KRAS, ERBB2, SMAD4, FBXW7, and CDKN2A [70–72]. Nakamura et al. [70] also identified ELF3 as a significantly altered gene in BTC, PRKACA, and PRKACB fusions were detected in single case of eCCA. Gallbladder cancers frequently exhibit EGFR and ERBB3 alterations. APOBEC-mediated mutational signatures are more prevalent in gallbladder cancer and eCCA [70]. A study by Jusakul et al. [73] identified four molecular clusters, underscoring how varying genetic, epigenetic, and environmental factors drive diverse molecular subtypes of CCA. Fluke-positive CCAs featured ERBB2 amplifications and TP53 mutations, while fluke-negative CCAs exhibited high copy-number alterations, PD-1/PD-L2 expression, epigenetic mutations (IDH1/2, BAP1), and FGFR/PRKA-related rearrangements. The whole-exome sequencing in the study by Grimsrud et al. [74] identified 53 candidate cancer genes with 123 nonsynonymous alterations common to two or more samples. Among these, 19% were novel to BTCs, including CNGA3, KRT28, and EFCAB5.

Analysis [69] of PSC-associated BTC samples using massive parallel sequencing revealed 247 nonsynonymous mutations and 89 copy number alterations (CNAs) across 30 of 42 targeted genes in 146 samples. Common genomic alterations were found in TP53, KRAS, CDKN2A, SMAD4, PIK3CA, CDKN2B, ERBB2, KDM5A/6A, and ROBO1, with less frequent mutations identified in genes such as FBXW7, TGFBR2, and BRAF. No FGFR translocations or BRCA1/2 mutations were identified, and only one IDH1 mutation was found in a single CCA case with typical small-duct/cholangiolar histomorphology. CNAs were primarily detected in CDKN2B, CDKN2A, and ERBB2. This study suggests [69] that PSC-BTC exhibits a phenotype similar to extrahepatic, large-duct bile duct cancer, regardless of anatomical location. This includes many intrahepatic tumors, which lacked the typical genomic alterations of iCCA such as IDH1/2 mutations and FGFR2 translocations. Instead, the intrahepatic tumors resembled eCCAs both histologically and molecularly.

### Predictive molecular biomarkers

As discussed in the previous section, significant differences exist in the genetic profiles regarding targetable mutations among anatomical subtypes of CCA. Mutations in FGFR2, IDH, and NTRK are important therapeutic targets in iCCA [75–77]. Mutational load and immune profiling suggest that hypermutated BTC cases with high immune checkpoint activity may benefit from targeted immunotherapies, particularly those with elevated immune checkpoint molecule expression, including PD-L1, in poor-prognosis clusters. Mody et al. [78] found that 8.6% of BTCs are PD-L1 positive, with the highest occurrence in gallbladder cancer (12.3%), followed by iCCA (7.3%) and eCCA (5.2%). PD-L1-positive tumors showed a significant increase in mutations of BRAF, BRCA2, RNF43, and TP53, as well as higher levels of biomarkers TOP2A, high tumor mutational burden (TMB high), and microsatellite instability (MSI-H). The study did not include assessment of possible risk factors. Given the molecular characteristics of PSC-associated biliary tract cancer, which align closely with eCCA, there is potential to explore targeted therapies similar to those used in this type of cancer. The consistent presence of genomic alterations such as TP53, KRAS, CDKN2A, and SMAD4 suggests that inhibitors targeting KRAS pathways or cell cycle regulators could be beneficial. Additionally, the overexpression of HER2/ERBB2 indicates that therapies like pertuzumab and trastuzumab, which target HER2, might prove effective [79]. As specific biomarkers for PSC-IBD-associated CCA have not yet been identified, integrating genomic profiling into future research could uncover new therapeutic targets, allowing for more precise and effective treatment strategies for this patient subset.

## Conclusion

In PSC-IBD patients, both CRC and CCA represent serious complications, with PSC and IBD recognized as independent risk factors for cancer development. Tumors in these patients often develop more rapidly, are frequently undetectable by standard endoscopy, and exhibit unique morphological, molecular, and prognostic traits, complicating early diagnosis. While numerous studies examine CRC and CCA in PSC or IBD separately, only a few addresses unique differences between sporadic, IBD-associated, and PSC-IBD-associated tumors. Given that the most of PSC patients also have IBD, findings on PSC-related tumors are often extended to PSC-IBD, reflecting shared inflammatory and pathophysiological pathways. However, distinct differences in microbial composition, immune responses, and molecular markers suggest unique tumor behaviors in PSC-IBD, influencing patient outcomes. Notably, predictive markers and actionable mutations for PSC-IBD-associated CRC and CCA remain limited, highlighting a need for targeted research to improve therapeutic options.In summary, CRC and CCA in PSC-IBD present unique diagnostic and therapeutic challenges, underscoring the need for specialized surveillance and exploration of specific biomarkers and mutations for targeted therapies.
